# Exercise intensity and energy expenditure of a multicomponent home-based training program: Xiangya hospital circuit training (X-CircuiT)

**DOI:** 10.3389/fpubh.2022.909766

**Published:** 2022-07-27

**Authors:** Peng Hu, Wenliang Zhang, Jeffrey W. Ripley-Gonzalez, Kangling Xie, Xun Gong, Zeng Cao, Yanan Shen, Baiyang You, Yaoshan Dun, Suixin Liu

**Affiliations:** ^1^Division of Cardiac Rehabilitation, Department of Physical Medicine and Rehabilitation, Xiangya Hospital of Central South University, Changsha, China; ^2^Cardiopulmonary Rehabilitation Center, Taihe Hospital, Affiliated Hospital of Hubei University of Medicine, Shiyan, China; ^3^National Clinical Research Center for Geriatric Disorders, Xiangya Hospital of Central South University, Changsha, China; ^4^Department of Cardiology, Hunan Provincial People's Hospital (The First-Affiliated Hospital of Hunan Normal University), Changsha, China; ^5^Division of Preventive Cardiology, Department of Cardiovascular Medicine, Mayo Clinic, Rochester, MN, United States

**Keywords:** home-based exercise, multicomponent exercise, X-CircuiT, exercise intensity, energy expenditure (EE)

## Abstract

**Introduction:**

Our previous studies showed that Xiangya Hospital Circuit Training (X-CircuiT) effectively improved physical fitness and reversed pre-frailty in community-dwelling older adults. This study aimed to explore the generalizability and applicability of X-CircuiT in different aged populations in the context of exercise intensity and energy expenditure.

**Methods:**

We prospectively recruited 72 community-dwelling sedentary adults, twelve adults divided into 6 age groups ranging from 20 to 80 years old and separated by decades. Cardiopulmonary exercise testing was performed to determine peak heart rate (HR_peak_). An individual HR-oxygen consumption regression equation was fit for each participant, and then a session of remote heart rate monitored X-CircuiT was performed. Exercise intensity (%HR_peak_) and energy expenditure of X-CircuiT among the six age groups were assessed. Further sub-analysis was conducted by dividing the participants by peak metabolic equivalent (MET) values, <5 METs, 5–7 METs, and more than 7METs to explore the relationship between maximum exercise capacity and exercise intensity of X-CircuiT.

**Results:**

The average %HR_peak_ of X-CircuiT for subjects in the 20–29, 30–39, 40–49, 50–59, 60–69, and 70–80 age groups were 54 ± 6, 59 ± 8, 60 ± 8, 62 ± 5, 66 ± 10, and 67 ± 13, respectively (*p* = 0.008); and the average energy expenditure was 121.9 ± 26.5, 123.3 ± 33.8, 129.2 ± 40.9, 130.9 ± 31.8, 146.8 ± 29.0, and 125.0 ± 28.4 kcal, respectively. The average %HR_peak_ for the warm-up, aerobic, acupoint patting, resistance, and stretching stages in overall subjects was 61 ± 9, 70 ± 10, 70 ± 10, 63 ± 9, and 57 ± 9, respectively. Furthermore, when subjects were divided by peak METs, it was found that the lower the peak METs, the greater the value of the relative exercise intensity indicators. The aerobic and acupoint stages of X-CircuiT could illicit a response of high intensity for those with peak METs <5, moderate intensity in those with peak METs of 5–7, and low-intensity for those with peak METs of more than 7.

**Conclusion:**

Xiangya Hospital Circuit Training followed the principle of low-intensity warm-up and medium-intensity training with multicomponent exercise training. It is classified as a moderate-intensity exercise for sedentary middle-aged and older adults, or those with a maximum exercise capacity of 5–7 METs, and is classified as a low-intensity exercise for young people.

## Introduction

Physical inactivity and increased sedentary time, are prominent factors in the etiology of cardiovascular (1) and metabolic diseases, as well as negatively affecting psychological well-being (2, 3). Conversely, routine exercise is effective for the prevention as well as treatment of multiple somatic and psychological health issues (3–6). Despite the overwhelming evidence and well-known benefits of exercise, however, most adults maintain relatively sedentary lifestyles. Moreover, as physical inactivity accounts for approximately 3.2 million deaths a year globally (7), there is an impetus to introduce potential methods to reduce this.

Barriers to initiating an exercise regime are multidimensional and include both internal and external reasons. The most commonly cited external factors often include perceived lack of time and lack of facilities, while internal factors include lack of training knowledge and lack of motivation/effort (8). Furthermore, newcomers to exercise may select singular forms of increased physical activity while perhaps lacking the awareness of the multinational guidelines and recommendations on further facets of the multiple components of physical exercise (9).

The onset of the coronavirus disease 2019 (COVID-19) has presented a new challenge. Lockdowns and travel restrictions have been established, substantially disrupting the physical activity and dietary habits of people (10). During quarantine, barriers to exercise, particularly accessibility to equipment and ease of use, have become ever more prevalent. The resulting increased sedentary time and limited physical activity have led to increased weight and worsening physical and psychological health across multiple populations, such as heightened depression and anxiety (11) among young adults and more so in the middle-aged and older adults (12, 13).

To overcome or mitigate these issues, community or home-based exercises have been promoted by clinicians and entities alike (14), including our team. The Xiangya Hospital circuit training (X-CircuiT) program is a multi-component exercise program developed by the cardiac rehabilitation team, based on the current multinational guidelines, such as components of cardiopulmonary, resistance, flexibility, and neuromotor exercise, as well as stretching (15). X-CircuiT has been used for the past 8 years. It was previously found to safely and effectively improve heart and lung function, muscle strength, and endurance in healthy adults (16) and could reverse pre-frailty in pre-frail older adults (17). Furthermore, due to its ease of practice and instructional video, X-CircuiT was found to have high accessibility and acceptability (17).

Our program consists of five distinct stages: warm-up, aerobic, acupoint patting, elastic band resistance, and stretching training. Within each of these stages, we included elements of flexibility, coordination, and balance and integrated elements of the traditional Chinese neuromotor exercise. Research has established that moderate aerobic exercise and resistance training bring marked improvements in cardiovascular health and mobility (18, 19), and can effectively improve muscle strength, gait function, balance, and quality of life; flexibility training can effectively restore range of motion in various joints and improve functional outcomes (20). Acupoint patting has shown promising results in the treatment of anxiety, depression, and stress disorder (21).

Despite evidence of the use of X-CircuiT, further research is required to elucidate the generalizability and applicability of X-CircuiT in different age and sex populations. Therefore, this study intends to evaluate the intensity and energy expenditure of the X-CircuiT, which may provide the basis for the proliferation of its use.

## Methods

### Subjects studied

**Figure 1:**
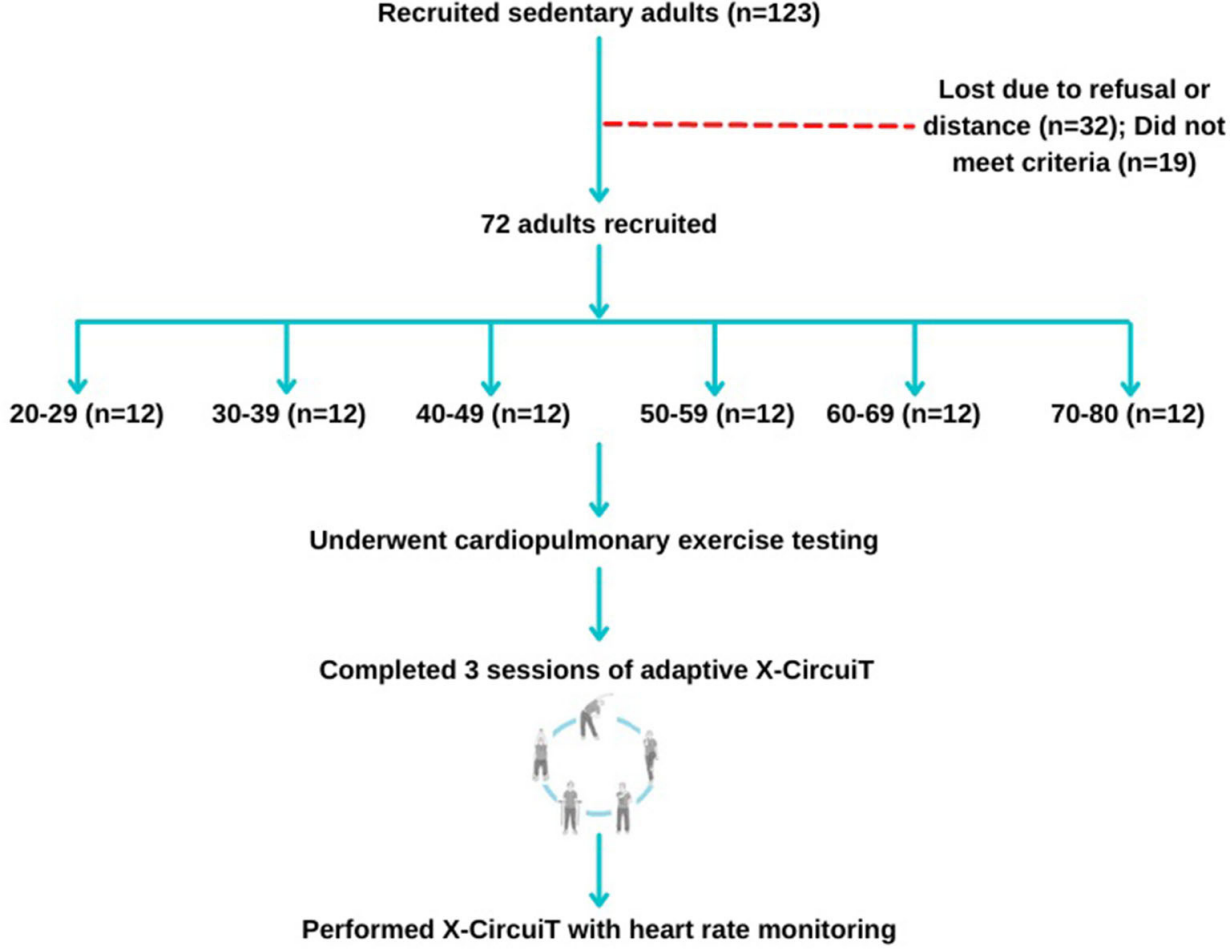
Participant recruitment, randomization, and study progress.

This cross-sectional study enrolled 72 volunteering sedentary adults from the Qing Yuan community, Changsha, China, between 1 August 2021 and 30 November 2021. The inclusion criteria were as follows: (1) age > 18 years; (2) sedentary adults [defined in this study as spending more than 8 h/day sitting or reclined (22)]; and (3) voluntarily signed informed consent and actively participated in the exercise program. Participants were excluded for any one of the following reasons: (1) the presence of any unstable clinical condition; (2)an inability to exercise or a condition that may interfere with the exercise performance; (3) myocardial infarction in the past 3 months; (4) comorbidities that may influence exercise physiological response or energy expenditure, such as hyperthyroidism; (5) signs of ischemia during exercise testing; (6) high risk of cardiovascular events or injury during community exercise; and (7) current participation in an ongoing exercise intervention. The flowchart of this study is displayed in Figure 1. Subjects were enrolled into one of six groups based on their age: 20–29, 30–39, 40–49, 50–59, 60–69, and 70-80 years old, with 12 adults per group.

Sample size calculation: The preliminary pre-experiment included 12 subjects aged 20–29 years and found that the standard deviation (SD) of exercise intensity (%HR_peak_) of X-CircuiT σ was 6 and the allowable error δ was 4 (95% *CI*/2). A sample size of 9 people would be needed to achieve an α of 0.05, according to the formula: *n* = (Z_1−α/2_ σ/δ) ^2^. This study was approved by the Ethics Committee of Xiangya Hospital, Central South University (No. 202009264). All subjects signed an informed consent form.

### Cardiopulmonary exercise testing

From August to November 2021, all participants performed a cardiopulmonary exercise test (CPET) 1 week before practicing X-CircuiT in Xiangya Hospital, Central South University, to obtain the peak heart rate (HR_peak_), peak oxygen uptake (VO_2peak_), and the individual regression equation of HR and VO_2_. The standard of procedure for performing CPET has been described previously (23, 24). The HR and VO_2_ data collected from the CPET were used to fit the individual regression equation of HR-VO_2_, according to which we calculated the VO_2_ during X-CircuiT. The reliability and validity of this method has been reported previously (25–27).

### X-CircuiT HR monitoring

A cardiac rehabilitation physician who was familiar with X-CircuiT and had relevant teaching experience was responsible for instructing X-CircuiT to the participants. After three sessions of adaptive training, participants were met in the Qing Yuan community gym. Here, participants were tasked to complete a set of standard actions in rhythm with the X-CircuiT DVD, during which the heart rate (HRex) was continuously monitored and recorded *via* a remote electrocardiogram monitoring system (DL-191, Good Friend, Shenzhen, China).

### X-CircuiT action plan

Xiangya Hospital Circuit Training consists of five stages lasting a total of 46 min. The instructional video and guide of X-CircuiT have been demonstrated previously (17). Briefly, each session began with a 4.5-min warm-up incorporating full coordinated upper and lower movement and dynamic stretching. This was followed by the aerobic stage, which along with the acupoint patting was divided into “elementary-level, intermediate-level, and advanced-level” sections based on the increased complexity of movement incorporated.

The aerobic stage (6.5-min) consists of 5 main movements, such as lateral steps, lateral cross-steps, knee-ups, forward lunges, and front steps. The Acupoint patting (6-min) involved coordinated aerobic movements along with open palm strikes directed at the Jian Jing (located on the shoulder) and Ba Liao (lower pelvic region), Ying and Yang meridians located on the inside and outside of the arms, respectively, Hegu (between the 1^st^ and the 2^nd^ metacarpal bones) and Houxi (when the fist is half-closed, the point is located in a depression proximal to the head of the fifth metacarpal bone), abdomen and lower back channels, and acupoints located in the leg.

The fourth stage of the circuit is the elastic band resistance training (15-min) targeting the large upper and lower limbs muscle groups, biceps, triceps, and quadriceps. The action sequence includes elastic band pull-aparts, front and lateral raises, elbow flexion/bicep curl, and elastic band lateral leg abductions. The elastic bands were color-coded, pink, orange, green, and blue (Sanctband elastic band), increasing tensile resistance in turn. Subjects were individually assigned an elastic band color based on the load-repetition relationship of resistance training, and we selected 50–60% of 1-RM, that is biceps curl to fatigue by repeating 17–20 repetitions. Valsalva movements were avoided during elastic band resistance training (17).

The final stage, flexibility training (14-min), focused on the stretching of major muscles, such as the pectoral muscle group, erector spinae, latissimus dorsi, trapezius, and major muscle groups in the leg. The main actions included dynamic and static stretching shoulder flexion, shoulder abduction, shoulder extension, and trunk stretch.

### Exercise intensity and energy expenditure of X-CircuiT

The common indicators of exercise intensity were HR and VO_2_, and their derivative variables, such as metabolic equivalents (METs), percentage of achieved HR to HR_peak_ (%HR_peak_), percentage of achieved VO_2_ to VO_2peak_ (%VO_2peak_), and percentage of HR reserve and VO_2_ reserve (%HRR and %VO_2_R). VO_2_ during the X-CircuiT (VO_2_ex) was calculated by the individual HR-VO_2_ equation, VO_2_ in L/min = coefficient × HRex in bpm + constant. The derivative variables of HR and VO_2_ were calculated by the following equations: (i) METs = VO_2_ex in L/min × 1,000/body weight in kg/3.5; (ii) %VO_2peak_ = (VO_2_ex/VO_2peak_) × 100%; (iii) %VO_2_R = (VO_2_ex - VO_2rest_)/(VO_2peak_ - VO_2rest_) × 100%; (vi) %HR_peak_ = (HRex/HR_peak_) × 100%; and (v) %HRR = (HRex - HR_rest_)/(HR_peak_-HR_rest_) × 100%. We followed the American College of Sports Medicine (ACSM) criteria to classify the level of exercise intensity (27, 28). Energy expenditure was calculated by the equation of calories = [METs × body weight in kg × 3.5]/200 × exercise time (29).

### Maximum exercise capacity and exercise intensity of X-CircuiT

For a secondary sub-analysis, participants were split based on their maximum exercise capacity to explore its relationship with the exercise intensity of X-CircuiT. Three groups were formed based on peak MET values, <5 METs, 5–7 METs, and more than 7 METs.

### Statistical analysis

Continuous variables, such as demographics, exercise intensity, and energy expenditure of participants are presented as mean ± standard deviation (SD), while categorical variables, such as sex, and whether overweight as a number (percentage). Differences between groups, divided by age, sex, and peak MET values, were analyzed using independent sample *t*-tests or analysis of variance (ANOVA) for continuous variables, while the chi-square test was used for categorical variables, according to the type of distribution. Statistical analyses were performed using the SPSS v. 25.0 (IBM Corporation, Chicago, IL, USA) and GraphPad Prism v.7.0 (GraphPad Software Corporation, California, USA) and statistical significance was set at α = 5%.

## Results

### Demographics and characteristics of subjects

The demographics and characteristics of the 72 subjects enrolled in the study are presented in Table 1. The difference between the six groups in HR_peak_, VO_2peak_, VO_2peak_ per kg, peak workload, and peak METs value was significant (*p* < 0.001 for each comparison), with a trend for gradual decrease with age (Table 1). The differences between the six groups in sex, weight, body mass index (BMI), HR_rest_, and peak respiratory exchange rate are displayed in Table 1. No significant difference was observed in blood pressure and peak VO_2_ pulse between the six groups.

**Table 1:** Demographics of the participants.

	**Total (*n* = 72)**	**20–29 yrs (*n* = 12)**	**30–39 yrs (*n* = 12)**	**40–49 yrs (*n* = 12)**	**50–59 yrs (*n* = 12)**	**60–69 yrs (*n* = 12)**	**70–79 yrs (*n* = 12)**	***P-*** **value**
Female, *n* (%)	41 (59)	6 (50)	9 (75)	8 (66)	7 (58)	6 (50)	6 (50)	0.75
Body weight, kg	61.8 ± 10.6	58.6 ± 8.4	60.1 ± 10.5	62.8 ± 12.5	65.9 ± 8.9	62.7 ± 11.2	60.1 ± 12.2	0.57
Height, cm	163 ± 9	165 ± 10	165 ± 10	163 ± 8	163 ± 9	161 ± 7	160 ± 9	0.70
BMI, kg/m^2^	23.2 ± 2.9	21.6 ± 1.5	22.0 ± 1.9	23.5 ± 3.2	24.7 ± 2.2	24.2 ± 4.0	23.5 ± 3.1	0.08
Overweight, *n* (%)	25 (35.7)	1 (8.3)	2 (16.7)	4 (33.3)	7 (58.3)	5 (41.7)	6 (60.0)	0.05
CPET parameters								
HRrest, bpm	72 ± 10	72 ± 10	75 ± 12	74 ± 11	75 ± 9	68 ± 7	68 ± 8	0.42
HRpeak, bpm	153 ± 22	170 ± 11	166 ± 19	163 ± 8	153 ± 17	135 ± 20	126 ± 20	<0.001
Peak VO_2_, L/min	1.50 ± 0.50	1.83 ± 0.61	1.48 ± 0.46	1.62 ± 0.59	1.45 ± 0.30	1.47 ± 0.42	1.07 ± 0.32	<0.001
Peak VO_2_, ml/kg/min	24.1 ± 6.2	30.6 ± 6.9	24.3 ± 4.1	25.4 ± 5.3	21.9 ± 2.7	23.6 ± 6.6	17.8 ± 4.3	<0.001
Peak METs	6.9 ± 1.7	8.5 ± 2.0	7.0 ± 1.2	7.2 ± 1.5	6.2 ± 0.8	6.7 ± 1.7	5.1 ± 1.2	<0.001
Peak RER	1.16 ± 0.09	1.15 ± 0.07	1.20 ± 0.09	1.15 ± 0.12	1.15 ± 0.10	1.18 ± 0.09	1.14 ± 0.06	0.61
Peak workload, watts	111 ± 39	144 ± 47	122 ± 43	118 ± 32	105 ± 27	92 ± 20	73 ± 25	<0.001

*BMI, body mass index; CPET, cardiopulmonary exercise testing; HR, heart rate; VO_2_, oxygen consumption; METs, metabolic equivalents; RER, respiratory exchange rate*.

### X-CircuiT heart rate response

**Figure 2:**
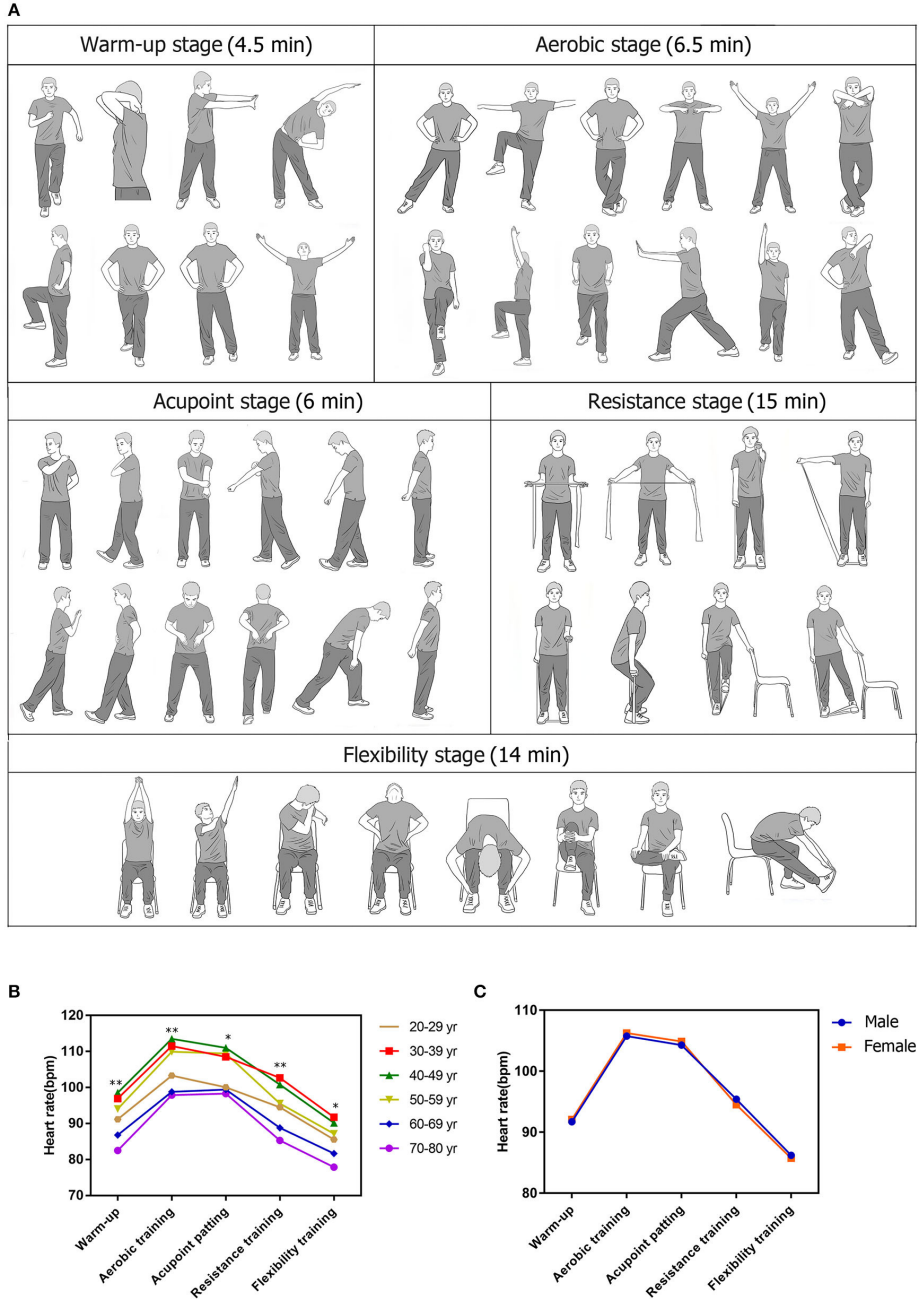
Actions, heart rate (HR) response of Xiangya Hospital Circuit Training (X-CircuiT). **(A)** shows an excerpt of X-CircuiT actions; **(B,C)** presents the heart rate response of X-CircuiT in the different age or sex groups, respectively. *Indicates the difference between groups *p* < 0.05; **indicates the difference between groups *p* < 0.01 (ANOVA) in **(B)**; An independent sample *t*-test in **(C)**.

Figure 2 shows the trend of HR response to X-CircuiT. HR gradually increased from the warm-up to the aerobic exercise stage, and then gradually decreased following the acupoint patting stage. The warm-up, aerobic, acupoint patting, resistance training, and flexibility training showed a heart rate of 92 ± 12, 106 ± 13, 105 ± 13, 95 ± 13, and 86 ± 11 beats per min (bpm), respectively. The average HR of the whole set was 95 ± 12 bpm, the HR fluctuated between 83 and 107 bpm, and the highest HR occurred at the 8 min and 30 s timepoint (107 ± 12 bpm), during the advanced-complexity part of the aerobic exercise stage, the lowest HR occurred at the 40^th^ min (83 ± 11 bpm), during the flexibility training knee raise. This trend remained true across age groups (Figure 2B) and sex (Figure 2C).

### X-CircuiT exercise intensity

**Figure 3:**
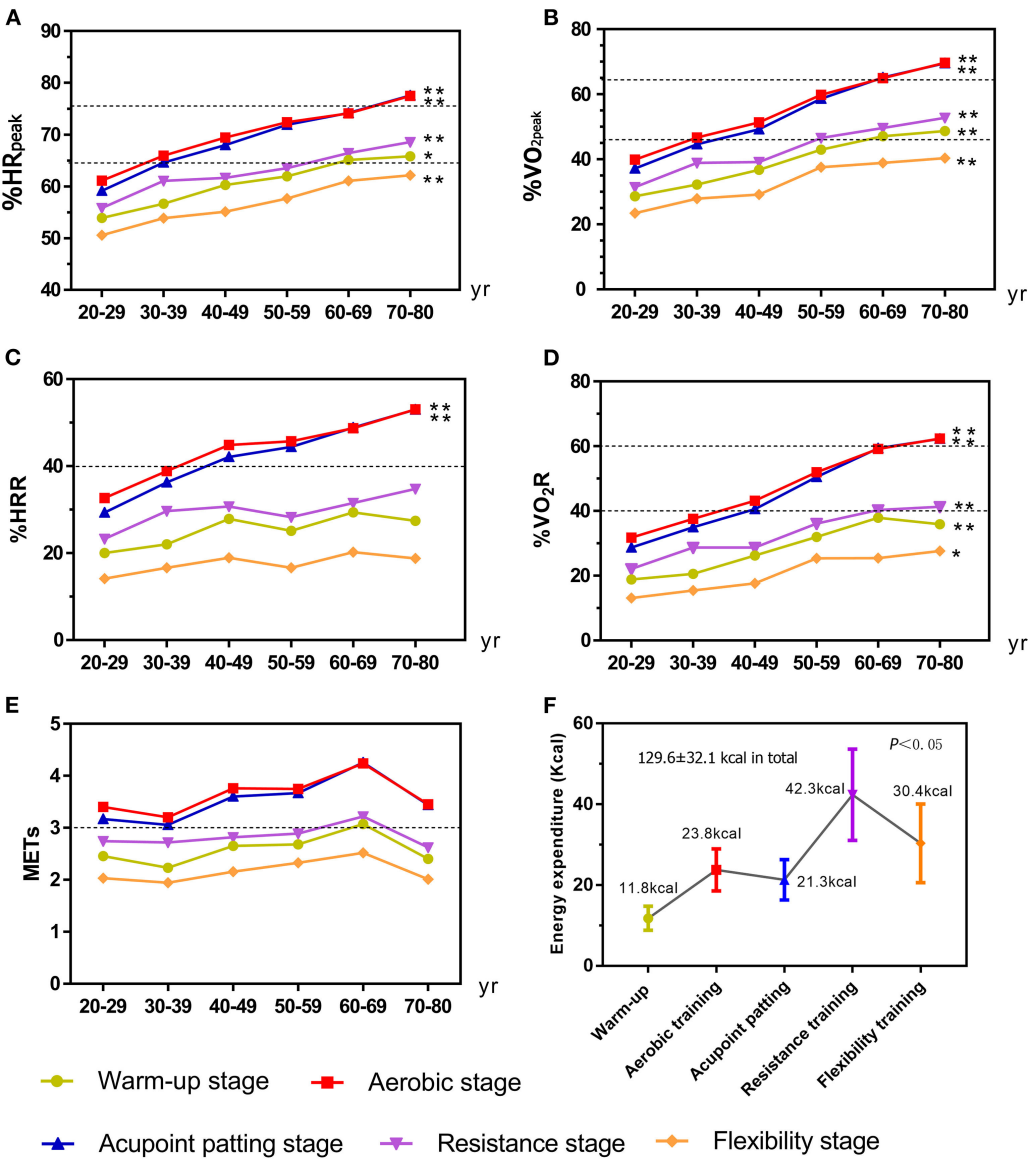
The exercise intensity and energy expenditure of the X-CircuiT program between different age groups. **(A)** Shows the peak heart rate percentage (%HR_peak_); **(B)** Shows the peak oxygen intake percentage (%VO_2peak_); **(C)** Shows the heart rate reserve percentage (%HRR); **(D)** Shows the oxygen uptake reserve percentage (%VO_2_R); **(E)** Shows metabolic equivalent (METs); and **(F)** Shows the energy expenditure of each stage and the whole set of the X-CircuiT. The dotted lines are the medium intensity exercise (lower line) or high intensity (higher line) exercise boundary. *Indicates the difference between groups *p* < 0.05; and **indicates the difference between groups *p* < 0.01 (ANOVA).

Oxygen utilization in L/min during the X-CircuiT was calculated by the individual linear regression equation previously shown (18, 19). Figure 3 shows that under the continuous activity of X-CircuiT, the aerobic exercise stage had the greatest intensity, followed by the acupoint patting stage, gradually falling in intensity thereafter with the flexibility training stages having the lowest exercise intensity. In addition, for the 40–80 age group, whether regarding absolute exercise intensity (METs) or relative exercise intensity (%HR_peak_, %VO_2peak_, %HRR, and %VO_2_R), the warm-up and flexibility stages were low-intensity exercises, while aerobic exercise and acupoint patting stages were of moderate-intensity. The absolute and relative intensities of each stage are presented in Figure 3. There were no statistical differences in absolute exercise intensity between the six age groups (*p* > 0.05), while the differences between the indicators of relative exercise intensity (%HR_peak_, %VO_2peak_, %HRR, and %VO_2_R) were statistically significant (*p* < 0.05). Figures 3A,B shows a comparison between the six groups in %HR_peak_ and %VO_2peak_, respectively. Each stage of X-CircuiT was statistically significant (*p* < 0.05), and the %HR_peak_ and %VO_2peak_ were observably greater with increased age. Figures 3C,D shows that the %HRR, and %VO_2_R in X-CircuiT's aerobic exercise and acupoint patting stages were significantly different with increased age (*p* < 0.05).

**Figure 4:**
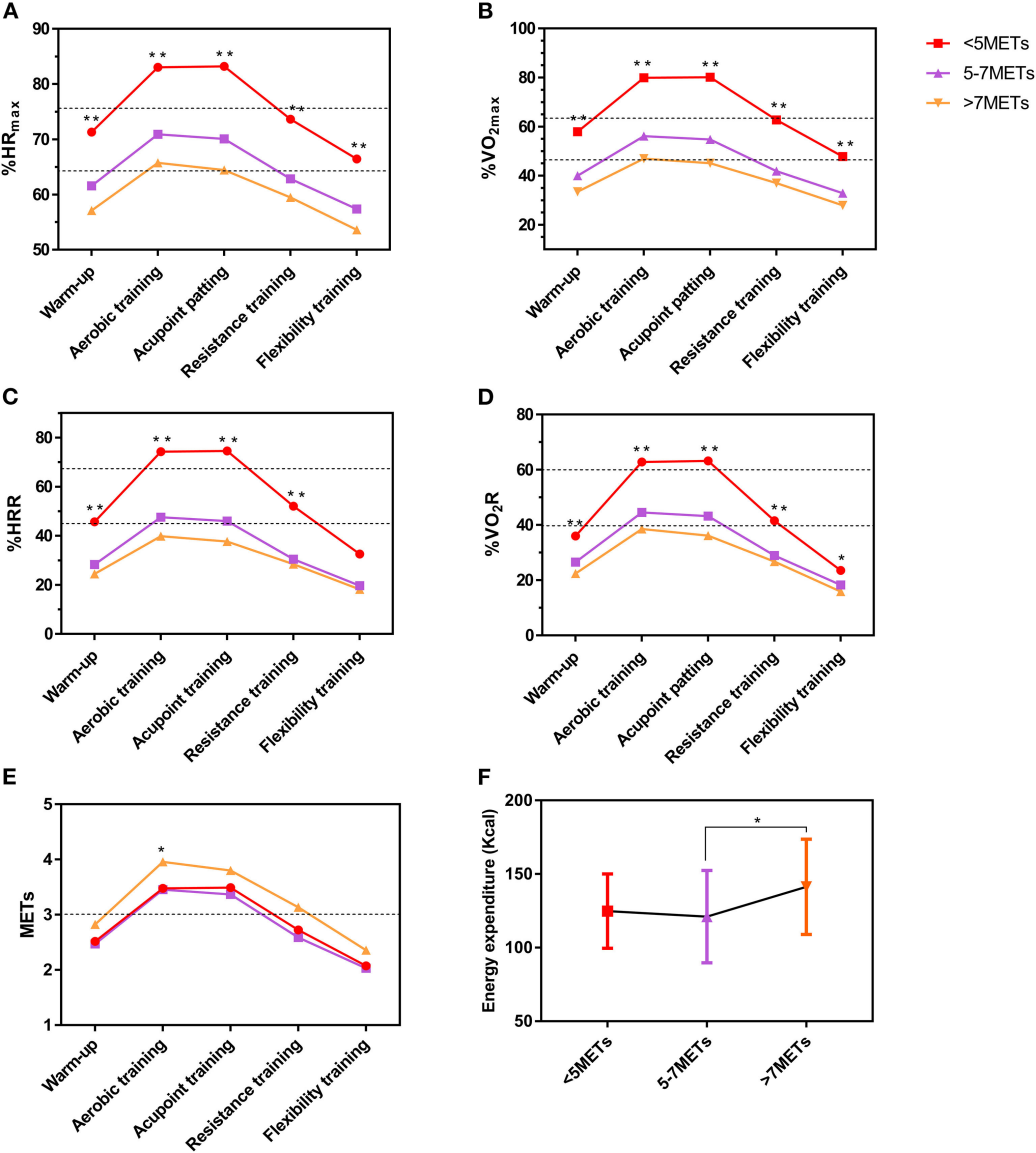
The exercise intensity and energy expenditure of the X-CircuiT program between different peak METs value groups. **(A)** Shows the peak heart rate percentage (%HR_peak_); **(B)** Shows the peak oxygen intake percentage (%VO_2peak_); **(C)** Shows the heart rate reserve percentage (%HRR); **(D)** Shows the oxygen uptake reserve percentage (%VO_2_R); **(E)** Shows metabolic equivalent (METs); and **(F)** Shows the energy expenditure of X-CircuiT. The dotted lines are the medium intensity exercise (lower line) or high intensity (higher line) exercise boundary. *Indicates the difference of the corresponding stage between groups *p* < 0.05; and **indicates the difference between groups *p* < 0.01 (ANOVA).

Figure 4 shows the relationship between the maximum exercise capacity of participants and the exercise intensity of X-CircuiT. We found that the lower the peak METs, the greater the relative exercise intensity indicators (%HR_peak_, %VO_2peak_, %HRR, %VO_2_R, and *p* < 0.05), while the aerobic training stage of the >7 METs group showed greater absolute exercise intensity than the other two groups (*p* < 0.05). The relative intensity and absolute intensity of aerobic training and acupoint patting stages of the <5 METs group (*n* = 11, 8 women), are all high-intensity, of the 5–7 METs group (*n* = 33, 25 women), moderate-intensity, and for the >7METs group (*n* = 28, 9 women), low-intensity.

**Figure 5:**
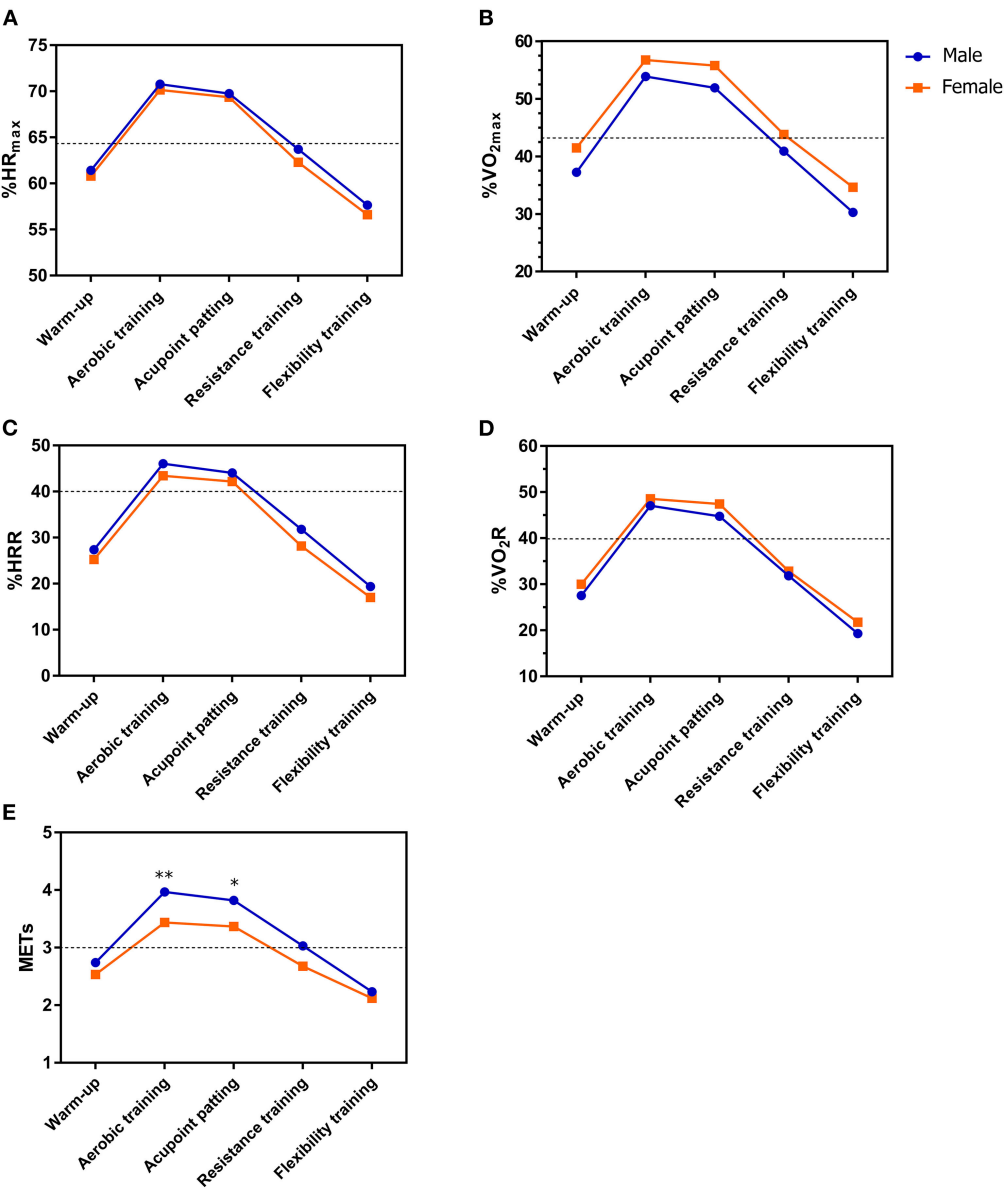
The exercise intensity of X-CircuiT in different sex groups. **(A)** Shows the peak heart rate percentage (%HR_peak_); **(B)** Shows the peak oxygen uptake percentage (%VO_2peak_); **(C)** Shows the heart rate reserve percentage (%HRR); **(D)** Shows the oxygen intake reserve percentage (%VO_2_R); and **(E)** Shows metabolic equivalent (METs). The dotted lines are the medium intensity exercise (lower line) or high intensity (higher line) exercise boundary. *Indicates the difference of the corresponding stage between groups *p* < 0.05; and **indicates the difference between groups *p* < 0.01 (an independent sample *t*-test).

We found absolute exercise intensity (METs) of X-CircuiT was greater among men than in women. However, no significant differences were found between sex in relative exercise intensity (%HR_peak_, %VO_2peak_, %HRR, and %VO_2_R) (Figure 5).

### Energy expenditure of X-CircuiT

As shown in Figure 3F, under the continuous activity involved when undertaking X-CircuiT, the energy expenditure of the warm-up, aerobic training, acupoint patting, resistance training, and flexibility training was 11.8 ± 3.0, 23.8 ± 5.2, 21.3 ± 5.0, 42.4 ± 11.3, and 30.4 ± 9.7 kcal, respectively. The energy expenditure of the overall X-CircuiT program was 129.6 ± 32.1 kcal.

## Discussion

Xiangya Hospital Circuit Training is a multi-domain exercise program that was previously shown to be accessible, effective, and safe in improving function in older adults (16, 17). It has an accompanying video and is tailored for practice at home or in the community. It has shown high acceptability in adults and may be a tool to improve sedentary behaviors and promote healthy lifestyles.

In the present study, we found X-CircuiT to be a moderate-intensity exercise for sedentary adults that elicits an energy expenditure and intensity similar to that of brisk walking or light dance, meeting the requirements of a low-intensity warm-up and cooldown and moderate-intensity aerobic training as recommended by multinational guidelines (28, 29). It can be concluded that the absolute exercise intensity and relative exercise intensity of the warm-up and flexibility training stages of the X-CircuiT are categorized as low-intensity exercise. Broken down into age brackets, the aerobic exercise and acupoint patting stages constitute moderate-intensity exercise in the middle-aged and older brackets, and low-intensity for young people. Furthermore, the X-CircuiT aerobic and acupoint patting stages are classified as moderate-intensity exercises for people with a maximum exercise capacity of 5–7 METs.

The absolute intensity of X-CircuiT is not related to age. However, the relative intensity of exercise is closely related to age, meaning that the older the age, the greater the relative intensity of exercise. This may be explained by the physiological effects of aging, where aerobic capacity is reduced, which is highly related to the training load, in that if the same form of exercise is completed, the lower the aerobic capacity, the greater the training load would be (29). To test this theory, participants were also grouped based on peak METs rather than age. As predicted, the relative intensities of those with lower peak METs were significantly higher than those who achieved greater peak METs.

Relative exercise intensity is more accurate than absolute exercise intensity and more suitable as a prescription for exercise in older populations, taking into account the difference in individual exercise capacity levels (29, 30). In addition, the absolute intensity of X-CircuiT was sex-dependent, and the absolute intensity of men was greater than that of women, while the relative intensity of exercise was sex-independent. This may be because men's weight and BMI is larger than women's, and their coordination is inferior to women's, all of which increase their exercise intensity.

The benefits of exercise are well known, but it takes a certain amount of exercise. The American College of Sports Medicine recommends that adults maintain and promote health by performing no <150–300 min of moderate-intensity aerobic exercise per week combined with muscle-strengthening activities two times a week (28). As such the basic recommendations are categorized by cardiorespiratory exercise, resistance exercise, flexibility exercise, and neuromotor exercise.

Therefore, according to the results of this study, and following the fundamentals of intensity, volume, and frequency, it is recommended to carry out X-CircuiT 7 times a week in which the aerobic and acupoint patting stages would need to be repeated two times or combined with other forms of moderate-intensity aerobic exercise (e.g., walking) to reach 150 min per week, and carrying out elastic band resistance training one time every other day.

### Limitations

This study has certain limitations. First, although this study calculated and collected a certain sample size based on the results of the previous pre-experiments, the sample size is still relatively small, and a larger sample size will be included in the later stages to more accurately measure the intensity and energy expenditure of X-CircuiT. Second, this study measures the intensity and energy expenditure of motion under the continuous movement of the program rather than individual stages, and the heart rate and oxygen consumption between the parts may affect each other. However, in clinical application, these can be applied as a whole, and the acupoint patting stage can be applied separately. So the later stage will measure the intensity and energy consumption of the X-CircuiT acupoint patting stage.

## Conclusion

Xiangya Hospital Circuit Training conforms to the principle of low-intensity warm-up and medium-intensity training and could be recommended as a community/home-based exercise program for sedentary adults. Therefore, X-CircuiT should be used as a regular exercise program for sedentary middle-aged and older adults or those with an exercise capacity of 5–7 METs, and can be used as supplementary training in young adults.

## Data availability statement

The raw data supporting the conclusions of this article will be made available by the authors, without undue reservation.

## Ethics statement

The studies involving human participants were reviewed and approved by Ethics Committee of Xiangya Hospital, Central South University (No. 202009264). The patients/participants provided their written informed consent to participate in this study.

## Author contributions

SL and YD conceived and were involved in securing funding for the study, had full access to all the data in the study, and had the final responsibility for the decision to submit for publication. PH performed project administration, data collection, and analyses. SL and YD supervised the writing of the original draft. WZ, KX, XG, ZC, YS, and BY provided assistance in coordinating the study and aided in data collection. JR-G was involved in writing—review and editing and visualization. All authors contributed to the article and approved the submitted version.

## Funding

This work was supported by the National Natural Science Foundation of China (Grant Numbers 82172549 to SL and 82002403 to YD) and the Hunan Development and Reform Commission Foundation of China (Grant Number [2012] 1521 to SL).

## Conflict of interest

The authors declare that the research was conducted in the absence of any commercial or financial relationships that could be construed as a potential conflict of interest.

## Publisher's note

All claims expressed in this article are solely those of the authors and do not necessarily represent those of their affiliated organizations, or those of the publisher, the editors and the reviewers. Any product that may be evaluated in this article, or claim that may be made by its manufacturer, is not guaranteed or endorsed by the publisher.
